# Utilization of a National Writing Challenge to Promote Scholarly Work: A Pilot Study

**DOI:** 10.7759/cureus.21935

**Published:** 2022-02-05

**Authors:** Angela Keniston, Maria Frank, Lauren McBeth, Ebrahim Barkoudah, Juliessa Pavon, Nidhi Rohatgi, Valerie Vaughn, Sanjay Bhandari, Marisha Burden

**Affiliations:** 1 Division of Hospital Medicine, University of Colorado School of Medicine, Aurora, USA; 2 Department of Medicine, Denver Health, Denver, USA; 3 Hospital Medicine Unit, Division of General Internal Medicine, Department of Medicine, Brigham and Women's Hospital, Harvard Medical School, Boston, USA; 4 Division of Geriatrics, Department of Medicine, Duke University, Geriatrics Research Education Clinical Center, Durham Veterans Affairs Health Care System (VAHCS), Durham, USA; 5 Division of Hospital Medicine, Department of Medicine, Stanford University School of Medicine, Stanford, USA; 6 Division of General Internal Medicine, Department of Internal Medicine, Division of Health System Innovation & Research, Department of Population Health Science, University of Utah School of Medicine, Salt Lake City, USA; 7 Division of Hospital Medicine, Department of Internal Medicine, Michigan Medicine, Ann Arbor, USA; 8 Division of General Internal Medicine, Department of Internal Medicine, Medical College of Wisconsin, Milwaukee, USA

**Keywords:** faculty advancement, scholarship, academic writing, academic hospitalist, writing challenge

## Abstract

Background

Hospitalists value mentorship and scholarly work, yet often struggle to find time and mentors amid busy clinical workloads.

Objective

To help catalyze writing for hospitalists nationally, we created a Writing Challenge, where we asked hospitalists to commit to the goal of writing 400 words a day, four days a week, for four weeks.

Methods

Prospective, programmatic evaluation with daily logs followed by a survey at the completion of the project. The four-week Writing Challenge occurred between June 7 and July 5, 2021. Email invitations to participate in the challenge were disseminated to peer networks, and the challenge was promoted using social media. Participants agreed to attempt to write 400 words per day, four days per week, for four weeks.

Results

Seventy-four individuals from 28 institutions registered for the Writing Challenge, with 36 (49%) participating in the challenge by logging their writing. Participants wrote an average of 4,372 +/- 4,324 words during the challenge. Sixty-eight percent of the participants reported that their amount of writing increased during the challenge and 50% of the participants stated they planned to publish their work, though many participants (46%) reported struggling to write each day.

Conclusions

The Writing Challenge is one way to generate increased writing and may result in increased scholarly output for academic hospitalists.

## Introduction

The field of Hospital Medicine (HM) has experienced rapid growth due to expanding clinical needs and duties [1-2]. However, HM has struggled in the academic sphere due to limitations in mentorship and time to focus on scholarly pursuits [3-5]. These struggles have likely been exacerbated by the frontline role of hospitalists in the current COVID-19 pandemic.

With peer-reviewed publications often serving as a measure of academic success, ensuring faculty are publishing is key to individual development, academic promotion, and advancement of the field. A recent study highlighted that the median number of publications for academic hospitalists was zero for the hospitalist faculty at the instructor and assistant professor levels [6]. Another study highlighted that academic promotion of successfully promoted hospitalists depended (in part) on traditional academic domains, including publications [7]. Many barriers exist for academic hospitalists, including a lack of mentorship for clinician-educators and researchers [8] as well as sufficient time to pursue scholarly activities [3].

Cultivating scholarship in HM will likely require traditional and non-traditional tactics given the nature of HM work. There are some suggestions that supporting a daily writing habit may increase productivity in writing [9-10], and some studies have suggested that writing groups and peer support networks may be helpful [11-12]. Therefore, to encourage writing among hospitalists, we piloted a four-week “National HM Writing Challenge” (i.e. Writing Challenge) in which we asked hospitalists to commit to writing goals as a way to facilitate their writing habits.

## Materials and methods

The study design was a prospective programmatic evaluation with an initial survey and daily logs followed by a survey at the completion of the project. This project was reviewed by the Colorado Multiple Institutional Review Board and was not deemed to be human subjects research (COMIRB 21-3631). Participants were informed of the plan to evaluate the program at the time of registration.

Writing challenge

The four-week Writing Challenge occurred between June 7, 2021, and July 5, 2021. Email invitations to this challenge were disseminated to peer networks by members of the Society of Hospital Medicine’s Research Committee. In addition, the Writing Challenge was promoted using social media (i.e. Twitter) where users were directed to a website for enrollment (https://bit.ly/3zKVHh3).

Any hospitalist or other medical professional interested in participating in the Writing Challenge was eligible to participate. There were no stipulations on type of writing and any type of writing was encouraged. The Writing Challenge had the following structure:

(a) At the time of registration, participants committed to writing 400 words per day, four days per week, for four weeks (or 16 of the 28 days to allow for flexibility with clinical work). This was chosen, as a previous pilot had included 500 words per day, five days per week for five weeks, and the feedback received was that this was not feasible for those with busy clinical schedules.

(b) Participants received weekly emails reminding them to write and log the number of words written and a blog post covering writing tips. The actual writing of participants was not submitted.

(c) To generate further interest in the Writing Challenge, a leader board, an outwardly facing section of the Writing Challenge website ranking participants by the quantity of writing (for those who gave permission to do so) was created and shared via weekly emails and social media posts.

(d) Each week, a 15-minute session was held for participants to discuss what was working well during the Writing Challenge and what was not. These sessions were held via Zoom. These sessions also served as an opportunity for participants to share writing tips and tricks with the other participants. Following the 15-minute session, participants could then write (off-line) for the remainder of the hour.

(e) Participants were assigned an accountability partner (i.e. writing partner) if they were interested. The writing partner was incorporated into the program to help build accountability with others and to help foster the habit and commitment of writing. There was no official mentorship component of the program beyond the option of a writing partner and weekly sessions that offered writing tips (as described above).

Data collection

Data were collected from the participants at various time points:

(a) At the time of enrollment in the challenge: Participants provided baseline information on their area of focus, how often they write, confidence in writing ability on a scale of 1 to 9 (with 1 indicating not confident at all and 9 being very confident), whether they would like a writing partner, their specialty, percent clinical fraction full-time equivalent (cFTE) (i.e. percent time spent in clinical work if applicable), years of practice, and demographics.

(b) At the end of each writing day: Participants were asked to log their quantity of writing using the Research Electronic Data Capture tool (REDCap) [13] hosted by the University of Colorado. The questions included participant’s name, date of writing, and type of writing (manuscript, project proposal, grant application, narrative/perspective piece, editorial, promotions dossier, medical education curriculum, clinical guidelines or pathways, blog post, professional talk, professional writing, and other) as well as the number of words written that day.

(c) At the completion of the challenge: Participants were sent a survey that included questions about whether or not their writing frequency increased, decreased, or remained unchanged, whether the participant plans to publish their work, if/how they struggled, the frequency of their writing, whether or not the work would result in any measurable outcomes, whether they completed the challenge, their level of confidence in writing (similar scale as above), and the best practices to complete the challenge. Additionally, participants were asked about their engagement with the program (assignment of a writing partner, attending virtual sessions, accessing the website and content, and what they would like to see in future iterations of the program). Lastly, they were asked if they would participate in another round of the writing challenge in the future.

Outcomes

Our primary outcomes included: (a) the number of words written per day and (b) completion of the writing challenge. To capture a tangible outcome, we chose the number of words instead of the time spent brainstorming about a project. Our secondary outcomes included: (a) self-reported change in the frequency of writing during the challenge and (b) self-reported scholarly work that resulted from the Writing Challenge.

Statistical analysis

All analyses were performed using SAS Enterprise Guide 8.2 (SAS Institute Inc., Cary, NC). Descriptive statistics were computed for words per day and survey questions. A paired t-test was used to compare the confidence in writing pre- and post-challenge. Data were assessed for normal distribution by visual inspection of histograms. Means and standards were estimated for continuous variables when approximately normally distributed; otherwise, medians and IQRs were estimated.

Qualitative analysis

Free text responses were summarized according to high-level themes that were derived by the research team. Exemplar quotes were selected for each of the high-level themes.

## Results

Seventy-four individuals from 28 unique institutions spanning 15 states and two countries registered for the Writing Challenge. Of the 74 registered individuals, 36 (49%) were considered participants, as they logged writing at least once during the challenge (Table 1). Participants’ (N=36) area of focus included medical education (47%), quality improvement (39%), research (47%), clinical practice (44%), clinical operations (19%), and other (19%). Eighty-six percent of participants were physicians. The mean percent cFTE for participants was 42% (+/- standard deviation of 37). Participants who registered for the challenge described their writing habits (pre-writing challenge) as 1 (3%) daily, 12 (33%) more than once a week, 7 (19%) weekly, 10 (28%) monthly, 6 (17%) every few months, and 0 (0%) never. The mean confidence level, on a scale of 1 to 9, with 9 being very confident, was 6. Fifty-five percent of the participants requested a writing partner.

**Table 1:** Demographics SD = standard deviation; % clinical full-time equivalent (FTE) = percent clinical effort *Of the 74 participants signed up, 38 individuals did not actively participate in the challenge (i.e. no entries into the daily logs, though they may have filled out the final survey).

	Registered to participate in the Writing Challenge, N = 74*	Participated in the Writing Challenge, N = 36 (49%)
To which gender do you most identify? N (%)		
Woman	49 (66)	26 (72)
Man	19 (26)	9 (25)
Prefer not to answer	2 (3)	1 (3)
Missing	4 (5)	0 (0)
To which race do you most identify? N (%)		
Asian Indian	12 (16)	6 (17)
Black or African American	2 (3)	1 (3)
Chinese	4 (5)	2 (6)
Korean	1 (1)	0 (0)
White	39 (53)	21 (58)
Other	9 (12)	4 (11)
Prefer not to answer	2 (3)	2 (6)
Missing	5 (7)	0 (0)
To which ethnicity do you most identify? N (%)		
Hispanic, Latinx, or Spanish Origin	5 (7)	2 (6)
South or Central American	1 (1)	0 (0)
Other	10 (14)	4 (11)
Area of Focus, N (%)		
Education	36 (49)	17 (47)
Quality Improvement	30 (41)	14 (39)
Research	37 (50)	17 (47)
Clinical	32 (43)	16 (44)
Clinical Operations	17 (23)	7 (19)
Other	16 (22)	7 (19)
Role, N (%)		
Physician	66 (89)	31 (86)
Advanced Practice Provider	1 (1)	0 (0)
PhD	1 (1)	1 (3)
Other	5 (7)	3 (8)
Specialty, N (%)		
Adult Medicine	63 (85)	31 (86)
Med/Peds	3 (4)	1 (3)
Family Medicine	1 (1)	1 (3)
Geriatric	2 (3)	0 (0)
Pediatric	1 (1)	1 (3)
Other	6 (8)	4 (11)
% Clinical FTE, Mean +/- SD	48 +/- 36	42 +/- 37
Years of practice, Mean +/- SD	11 +/- 8	10 +/- 7
US Geographic Region, N (%)		
Northeast	5 (7)	3 (8)
Southwest	5 (7)	2 (6)
West	34 (46)	19 (53)
Southeast	5 (7)	3 (8)
Midwest	12 (16)	6 (17)
Not applicable	1 (1)	0 (0)
Missing	12 (16)	3 (8)
Frequency of writing, N (%)		
Daily	4 (5)	1 (3)
More than once a week	18 (24)	12 (33)
Weekly	19 (26)	7 (19)
Monthly	19 (26)	10 (28)
Every few months	2 (3)	6 (17)
Never	12 (16)	0 (0)
Confidence in writing ability (1 being not confident at all, 9 being very confident), Mean +/- SD	5 +/- 2	6 +/- 2
Reports having writing team or partner for this challenge, answer indicates "yes", N (%)	20 (27)	14 (39)
Would like to be assigned a partner for this challenge, answer indicates "yes", N (%)	27 (53)	12 (55)

The mean number of logged entries for those who entered the Writing Challenge was 8 (standard deviation of +/- 6) with the goal for the Writing Challenge being 16. A sum total of 157,402 words were written, with an average of 4,372 +/- 4,324 words written per participant (goal was 6,400 words to complete the challenge). The median number of words written per participant was 2,815.5 (interquartile range (802, 6,972.5). The most common types of writing logged included: manuscripts (56%), personal writing (44%), and narrative/perspective pieces (28%) (Table 2).

**Table 2:** Daily writing log results SD = standard deviation *Includes free writing, advocacy letters, letters of support/nomination/recommendation, abstracts, dissertation, and article reviews

	N = 36
Number of writing sessions logged, Mean +/- SD	8 +/- 6
Total number of words logged, Mean +/- SD	4,372 +/- 4,324
Number of different types of writing logged, Mean +/- SD	3 +/- 2
Types of writing logged, N (%)	
Manuscript (any scholarly manuscript for a peer-reviewed journal)	20 (56)
Personal writing	16 (44)
Other*	12 (33)
Narrative/perspective piece	10 (28)
Professional talk	9 (25)
Project proposal	8 (22)
Grant application	6 (17)
Promotions dossier	6 (17)
Med-Ed curriculum	5 (14)
Editorial	4 (11)
Blog post	2 (6)
Clinical guidelines or pathway, order sets	1 (3)

The post-survey had a response rate of 38% (28 individuals of the 74 who registered) (Table 3). Sixty-eight percent of respondents stated their writing increased during the challenge, 29% stated writing was unchanged, and 4% wrote less. Fifty percent of respondents stated they plan to publish the work. Forty-six percent of the respondents stated they struggled to find time to write each day. Three participants (11%) reported completing the Writing Challenge, 10 (36%) thought they completed the challenge but were not sure, and 15 (54%) reported not being able to complete the challenge. Writing confidence at the beginning of the challenge was 6.0 +/- 1.8 and at the end of the challenge was 6.4 +/- 1.4, p-value = 0.178. Qualitative themes with exemplar quotes about the feasibility of the challenge, reasons to participate in another challenge, and additional feedback are shown in Table 4. Respondents felt that virtual sessions with features, including speakers focusing on brief tips or best practices, would be helpful. Of the 29% (N=8) of participants that reported being assigned a writing partner, 38% reported meeting with their writing partner at least once. The Writing Challenge website was accessed by the majority of post-survey respondents with the majority finding the site and shared resources helpful.

**Table 3:** Hospital Medicine National Writing Challenge Program Evaluation Survey SD = standard deviation *Includes curricula, narrative/perspective piece, blog post, professional talks, professional writing (e.g., letters of support/nomination/recommendation)

Experience during writing challenge (select all that are true), N (%)	N = 28
My writing increased during the challenge	19 (68)
My writing was unchanged during the challenge	8 (29)
I actually wrote less	1 (4)
I plan to publish some of the work I wrote	14 (50)
I do not plan to publish any of the work I wrote	1 (4)
I struggled to find topics to write on each day	2 (7)
I struggled to find time to write each day	13 (46)
Frequency of writing during the challenge, N (%)	
Always	0 (0)
Often	16 (57)
Sometimes	8 (29)
Rarely	4 (14)
Never	0 (0)
Completion of writing challenge, N (%)	
Yes	3 (11)
No	15 (54)
I think so, but I'm not entirely sure.	10 (36)
Confidence in writing, Mean +/- SD	
After having completed the writing challenge, how confident are you in your writing ability? (1 being not confident at all, 9 being very confident)	6 +/- 1
Website, N (%)	
Did you access the Hospital Medicine National Writing Challenge website (response = yes)	23 (82)
Please select the website pages you viewed (please select all that apply)	
Writing blog, N (%)	11 (39)
Extremely useful	3 (27)
Very useful	4 (36)
Moderately useful	4 (36)
Slightly useful	0 (0)
Not at all useful	0 (0)
Resources	9 (32)
Extremely useful	1 (11)
Very useful	7 (78)
Moderately useful	0 (0)
Slightly useful	1 (11)
Not at all useful	0 (0)
Leader board	18 (64)
Extremely useful	1 (6)
Very useful	3 (17)
Moderately useful	8 (44)
Slightly useful	4 (22)
Not at all useful	2 (11)
None of the pages were useful	1 (4)
Did your participation in this challenge result in any of these measurable outcomes (please select all that apply), N (%)	
Manuscript (any type) completion/submission(s)	9 (32)
Proposal completion	6 (21)
Promotions dossier	4 (14)
Grant	0 (0)
Other measurable outcomes*	7 (25)
None of the above	9 (32)
Would you participate in another round of the Hospital Medicine National Writing Challenge? N (%)	
Definitely	15 (54)
Probably	11 (39)
Possibly	0 (0)
Probably not	2 (7)
Definitely not	0 (0)

**Table 4:** Exemplar quotes on the feasibility of the challenge, reasons to participate in another challenge, and any additional feedback

Topics	Themes/Quotes
Why or why not would you participate in another challenge?	Time/Timing
	Didn't meet my own expectations this time so would love to try again.
	I think it's a great idea, the timing just didn't work out for me this time as it was a busy month on service.
	It forced me to think about the act of writing more deliberately and for shorter chunks of time than I often do.
	Accountability/motivation
	It kept me accountable to write regularly.
	It was a good push and I wrote with others in my section.
	It was motivating to feel a part of a broader effort to increase writing, generally. I wish that I had participated in the virtual writing sessions, however was not so confident at being able to offer something - but would like to try again!
If no, why not? (i.e. what would make the challenge more feasible)	Prompting
	I didn’t feel external accountability/ motivation. The partnering was not really implemented. There were no daily reminders.
	Competing obligations
	It was a bad time - started when I was on service, then on vacation and then on service again. Need to do this when I have a freer window.
	This was a really tough time of year with turnover of academic year and graduating residents/new interns - I wanted to but literally could not find time as was working 11-hour days!
	This was a difficult time to focus on writing.
	Timing was just rough with lots of clinical and other work.
	My workload was too high during this time period and this challenge fell off my radar. Happy to try again next time though!
Please share any additional feedback.	Positive experience
	I thought it was a very positive overall experience. I finished one manuscript draft (already rejected so need to revisit), revisions on another (where I am NOT the first/senior author so had less responsibility, which has been accepted), two large presentations, and two letters of recommendation, as well as some work on a medical school curriculum. Thanks for organizing!
	This is a really neat project that stands to bring together writers far-flung across the country. It is especially appropriate to the times in which we live, when travel and other means to interface personally have been limited by the pandemic. Even in the absence of direct person-to-person contact, the project creates the stirrings of connection and commonality among health care professionals that are, at least in one manner, like-minded.
	Helpful strategies
	May be helpful to ask participants to block those 4x weekly times at the beginning of the month and to name at least two writing efforts. I had it as a to-do for each day of the month so was easier to de-prioritize.
	For the confidence question, to be clear, I think I was around the same, so it's not really that the writing challenge increased my confidence in my writing. It did help me be more self-aware of how much I was writing per week. Also, for the "assigned a partner" question, I paired up with a partner, but they were not "assigned" to me.

## Discussion

In its inaugural attempt, the Writing Challenge appeared to be successful. The Writing Challenge drew a diverse group of hospitalists and other professionals to commit to writing; approximately half of whom documented writing at least once during the challenge and two-thirds of whom reported increasing their writing during the challenge. The national Writing Challenge model may be one way to encourage writing and thus increase academic productivity in HM.

Previous work has highlighted that hospitalists are generally younger and often lack mentorship and the field has continued to struggle with how to mentor and build scholarly work [3]. The Writing Challenge represents one way to build accountability and increase writing output for work that could be published in the future. The Writing Challenge participants reported that finding time to write was very challenging, particularly when on clinical service. Previous work has highlighted the challenge between balancing clinical time and scholarly work [14] and we suspect the pandemic only further increased this challenge.

We chose the words written per day as one of our outcome measures. A recent study showed that although increases in weekly word counts did not necessarily reflect the manuscript quality or equate with manuscript submission, the incremental progress represented success over many frequently cited barriers (i.e. lack of time, workload demands, perfectionism, environmental distractions, and lack of experience, etc.) [15].

Our study has several limitations. The results reflect self-reports through surveys and did not have a control group. We did not verify the written work, nor judge the quality of the work as the goal was to build a daily writing habit. The participation rate was variable and likely impacted by the ongoing pandemic. The mean percent clinical effort was 48% and thus the participants represented a group of people who reported relatively low clinical effort, though this varied from no clinical effort to 100% clinical effort. It may be that participants that had a lower clinical effort had more time to write than those with higher clinical effort. A large proportion of participants were researchers and thus the results may be skewed. Over half of the participants were not able to complete the challenge. This project was previously piloted at a single institution with a goal of 500 words per day for five days per week. The feedback suggested that a lower word requirement should be attempted; however, even with a further reduction of the word requirements, participants struggled with the challenge. Future iterations could include daily email reminders, however, this must be balanced with too many communications. Additionally, the weekly meetings were informal; adding more structure to those sessions could be considered in future iterations of the program. Additional efforts at including more junior participants should be considered. In addition to the accountability partner, future program considerations could include offering mentorship to participants that report lower writing confidence levels. The Writing Challenge did require some administrative time to coordinate the program. We did not assess whether participants were in academic or community health care settings and thus it is unclear as to the impact of the Writing Challenge in these different settings. We did not assess academic rank, however, those that participated had approximately 11 years of experience, indicating more experienced participants though the range of experience was wide. It is unclear if the increases in writing that occurred during the challenge are sustained and thus future studies could include assessing writing habits at three or six months post completion of a writing challenge. Lastly, there are other barriers to writing leading to published work such as publication costs for open access journals.

There are also several strengths. We believe this is one of the first descriptions in the HM literature of a writing challenge and the outcomes of such a project. Sixty-six of those registered were women and 72% of those that participated in the Writing Challenge were women. Additional study should be considered, given this could be a tool to help address gender disparities in authorship [16]. We believe this represents a low-cost way of generating interest in writing and scholarship, and it has important implications for a scalable tool to increase academic output and could be utilized in other settings such as for trainees.

## Conclusions

The Writing Challenge led to increases in writing, with half of the participants stating that they plan to publish their work. It also helped increase confidence in writing. While hospitalists struggle with finding time to write because of clinical demands, the Writing Challenge represents a low-cost way of fostering interest and accountability in writing that may result in increased scholarly output.
